# A pilot randomized controlled trial of a multicomponent intervention to improve sexual function in female breast cancer survivors

**DOI:** 10.3389/fonc.2026.1895188

**Published:** 2026-09-09

**Authors:** Noël M. Arring, Carolyn Lafferty, Jeanne Carter, Deborah Marshall, Jennifer Barsky Reese

**Affiliations:** 1 College of Nursing, University of Manitoba, Winnipeg, MB, Canada; 2 College of Nursing, University of Tennessee, Knoxville, TN, United States; 3 Department of Dermatology, Michigan Medicine, University of Michigan, Ann Arbor, MI, United States; 4 Gynecology Service, Department of Surgery, Memorial Sloan Kettering Cancer Center, New York, NY, United States; 5 Department of Psychiatry, Memorial Sloan Kettering Cancer Center, New York, NY, United States; 6 Department of Radiation Oncology, Icahn School of Medicine at Mount Sinai, New York, NY, United States; 7 Department of Population Health Science and Policy, Icahn School of Medicine at Mount Sinai, New York, NY, United States; 8 Cancer Prevention and Control Program, Fox Chase Cancer Center, Philadelphia, PA, United States; 9 Department of Social and Behavioral Sciences; Temple University College of Public Health, Philadelphia, PA, United States

**Keywords:** body image, breast cancer, genitourinary syndrome of menopause (GSM), sexual health, women’s health

## Abstract

**Introduction:**

Sexual health challenges after breast cancer commonly include physical symptoms of genitourinary syndrome of menopause (GSM) (e.g., vaginal pain) and psychological problems (e.g., negative body image) necessitating interventions that address both issues.

**Objective:**

To assess the feasibility of a multicomponent intervention of hypnotic relaxation and vaginal moisturizer to improve GSM, sexual desire, and body image in female breast cancer survivors.

**Methods:**

This two-arm pilot randomized controlled trial tested an 8-week self-administered multicomponent intervention, hypnotic relaxation intervention (HRI) with vaginal moisturizer, compared to a vaginal moisturizer-only group (VMO). All participants used vaginal moisturizer daily in weeks 1 and 2. In weeks 3–8, vaginal moisturizer use decreased to every other day. In addition to vaginal moisturizer, HRI participants added hypnotic relaxation 3 times per week in weeks 3–8. Eligible women reported vaginal or vulvar dryness and/or pain with sexual activity, negative body image changes, and/or decreased sexual desire.

**Main outcomes and measures:**

The primary outcomes were accrual, retention, and adherence rates. Preliminary intervention effects on sexual function were assessed using the Sexual Function Inventory (FSFI) and PROMIS Sexual Function and Satisfaction V2 (SexFS V2) as well as on body image using the Breast Cancer Impact of Treatment Scale (BITS) at baseline and 8 weeks.

**Results:**

Thirty participants were randomized to HRI (*n* = 14) or VMO (*n* = 16); all completed the study. Adherence was excellent for both HRI (92%) and VMO (90%). Sexual desire, body image, and GSM-related outcomes (lubrication and pain) improved for both groups. Small to moderate effects were found in the change scores favoring the HRI group compared to the VMO group for SexFS V2 interest (*d* = 0.37) and body image (*d* = 0.42). In the full sample, large effects were found for an increase in lubrication, *d* = 1.50 (0.97, 2.02), and a reduction in pain, *d* = 0.81 (0.39, 1.22), on the FSFI.

**Conclusions and relevance:**

This randomized controlled pilot trial supported the feasibility of hypnotic relaxation and vaginal moisturizer for female breast cancer survivors with sexual problems. Hypnotic relaxation coupled with vaginal moisturizer appears to be a promising intervention, particularly for improving body image, and a larger, well-powered study is needed to confirm these findings.

**Trial registration:**

ClinicalTrials.gov, Identifier NCT05692960.

## 1 Introduction

Sexual health issues impact between 30% and 80% of women with a history of cancer (1). According to the World Health Organization, sexual health is core to human existence, contributes to overall wellbeing, and is a fundamental human right (2). A study of 7,895 female breast cancer survivors found that 78% reported at least one of the following: negative body image, decreased sexual functioning, less sexual enjoyment, and sexual inactivity (3). Sexual health concerns, including genitourinary syndrome of menopause (GSM), decreased sexual desire, and negative body image, can be long-term side effects for many breast cancer survivors (1, 4–7). According to Bober and Varela’s biopsychosocial framework of cancer-related sexual problems, sexual health is shaped by biological factors (e.g., hormonal changes, pain, vaginal dryness), psychological factors (e.g., emotions, body image, self-efficacy), interpersonal dynamics (e.g., relationship quality, fear of intimacy), and broader social and cultural contexts (e.g., religious beliefs, societal norms) (8).

Consistent with the biopsychosocial framework of cancer-related sexual problems, GSM symptoms, including vaginal dryness and dyspareunia, are consistently identified as key predictors of sexual functioning and satisfaction among female cancer survivors (4, 5 , 9, 10). Estrogen plays a critical role in maintaining vaginal tissue integrity. Cancer treatments that cause estrogen deprivation often lead to GSM with symptoms like vulvovaginal dryness, pain during sex, and increased risk of infections, all of which can negatively impact sexual satisfaction and interest (11). Within this biopsychosocial framework of cancer-related sexual problems, biologic domains (e.g., GSM symptoms) and psychological domains (e.g., body image and sexual desire) are conceptualized as interacting and mutually reinforcing. This underscores the importance of addressing GSM. Furthermore, a recent secondary analysis of a study evaluating bupropion as a treatment for sexual desire in female cancer survivors found that lubrication and pain were predictors of sexual desire, supporting this relationship (12). Additionally, over 93% of the female cancer survivors in the study had vaginal dryness. Together, these findings highlight the need to include the first-line treatment for vaginal dryness, non-hormonal vaginal moisturizers (13), in sexual health intervention studies for female cancer survivors (12).

Within the biopsychosocial framework of cancer-related sexual problems, sexual desire and negative body image, two psychological factors, are key predictors for female sexual health (14). Sexual desire in female cancer survivors is a multifactorial concept influenced by vulvovaginal health, intimate relationships, and body image (15). Negative changes in body image, such as feeling flawed, sexually unattractive, or feeling a loss of femininity, are among the complex issues surrounding sexual health for survivors of breast cancer (5). Because these concerns involve both psychological and embodied experiences, mind–body interventions may be particularly well suited to address them. Hypnotic relaxation is a mind–body intervention that uses the interventionist voice to place the person in a trance-like state while making suggestions for relaxation (16). Hypnotic relaxation tailored for sexual health has shown promise for improving body image and sexual satisfaction in cancer survivors (17, 18). Yet, given the multifactorial and interrelated nature of sexual health, there is a clear need for interventions that address multiple domains of sexual health concerns (19, 20). In this study, vaginal moisturizer and hypnosis were designed to target complementary domains of female sexual dysfunction, consistent with a biopsychosocial framework. The vaginal moisturizer addresses genitourinary symptoms (GSM), which are a key biologic barrier, while hypnosis targets psychological factors such as sexual desire and body image. Together, these components are intended to address interacting mechanisms that contribute to sexual dysfunction in female cancer survivors.

Therefore, in this feasibility study, we tested an 8-week self-administered multicomponent intervention that consisted of hypnotic relaxation intervention (HRI) with vaginal moisturizer compared to vaginal moisturizer only (VMO) as a treatment for GSM, body image, and sexual desire. The primary outcomes were accrual, retention, and adherence. We also examined preliminary intervention effects on sexual function using the Female Sexual Function Inventory (FSFI) and PROMIS SexFS V2 as well as on body image using the Breast Cancer Impact of Treatment Scale (BITS) to inform a larger trial.

## 2 Materials and methods

### 2.1 Study design

This two-arm pilot randomized controlled trial assessed the feasibility of an 8-week multicomponent intervention HRI compared to VMO as a treatment for GSM, negative body image, and low sexual desire among breast cancer survivors. The study was conducted at the University of Tennessee in Knoxville (UTK). The UTK IRB approved the study (UTK IRB-22-07195-XP), and it was registered with ClinicalTrials.gov (NCT05692960). Participants provided written informed consent. After attaining consent, a study coordinator randomized participants 1:1 using a random number generator. No participants or study personnel were blinded to group allocation. After study completion, the VMO group was offered HRI.

### 2.2 Sample

As a pilot study, a sample of 30 participants was deemed appropriate for the objectives of assessing feasibility outcomes, measuring sensitivity, and estimating effect sizes that can be used for the development of a larger study (21). Participants were recruited from the community and nationally through social media and ResearchMatch.org (volunteer database). Thirty participants were enrolled between February 2023 and December 2023. We included partnered adult women who had completed primary treatment for stage I, II, or III breast cancer at least 3 months, but not more than 5 years before enrollment. Eligible participants had to be physically able to engage in sexual activity, have vaginal or vulvar dryness and/or pain with sexual activity, and report one of the following since cancer diagnosis: (1) negative changes in body image or (2) low sexual desire. Adjuvant endocrine therapy or HER2-targeted therapy was permitted. Key exclusion criteria included a history of sexual abuse, psychiatric disorder, or use of estrogen.

### 2.3 Intervention

#### 2.3.1 Vaginal moisturizer only

Participants were directed to use a non-hormonal bioadhesive vaginal moisturizer applied to their vagina and vulva every night during weeks 1 and 2 and every other night during weeks 3 to 8 (13).

#### 2.3.2 Hypnotic relaxation intervention

In addition to using the vaginal moisturizer as described above, the HRI group used hypnosis recordings starting in week 3 consisting of three different audio recordings. Recordings were introduced sequentially with one recording assigned per 2-week period. Participants were instructed to listen to the assigned recording three times per week for 2 weeks. Each recording lasted approximately 20 min. The three recordings included hypnotic inductions that built upon each other. The first recording included suggestions for relaxation, wellness, and confidence; the second added suggestions for body image; and the third addressed sexual desire.

### 2.4 Measures

#### 2.4.1 Feasibility benchmarks

*A priori* feasibility benchmarks were ≥60% accrual rate, ≥80% retention rate, and an ≥80% adherence rate for each intervention (22, 23). Participants were considered retained if they completed study measures in week 8. Adherence for vaginal moisturizer was assessed in biweekly calls. Adherence to HRI was assessed with practice logs.

#### 2.4.2 Preliminary intervention effects

Sexual function and satisfaction were assessed using the FSFI and PROMIS SexFS V2. The FSFI measures the major domains of sexual functioning including desire, lubrication, and pain. Higher scores indicate better sexual function. The FSFI has strong internal reliability with Cronbach’s alpha over 0.9 (24). The SexFS V2 measures sexual activities, symptoms, functioning, and sexual experiences (25). Higher scores indicate better sexual function for included measures of satisfaction, interest, and lubrication. Raw scores were converted to *t*-scores, where the mean [standard deviation (SD)] = 50 (10) in referent population (25). The measure has good reliability with Cronbach’s alpha scores from 0.85 to 0.98 (25).

Body image was assessed using the BITS, which measures body change stress with scores ranging from 0 to 65. Higher scores indicate worse body change stress. The scale has good reliability with Cronbach alphas over 0.90 (26).

Adverse events (AEs) were assessed in biweekly phone calls using the Common Terminology Criteria for Adverse Events (CTCAE) version 5 (27). Vaginal moisturizer potential expected AEs were vulvovaginal irritation, rash, burning, and infection. HRI potential expected AEs were agitation and anxiety.

### 2.5 Data analysis

All data were summarized using descriptive statistics (mean, SD, percentage, frequency). Mean differences between study arms were compared using *t*-tests or chi-square analyses for all demographic and outcome variables. Mean difference Cohen’s *d* (*d*) effect sizes were calculated from baseline to week 8 for all exploratory outcome measures. IBM SPSS statistical software (version 27) was used for all analysis. All participants enrolled and randomized were included in the analysis according to their study assignment. Missingness was assessed using tests for missing completely at random (MCAR), and mean substitution was applied for variables meeting MCAR assumptions.

## 3 Results

**Figure 1:**
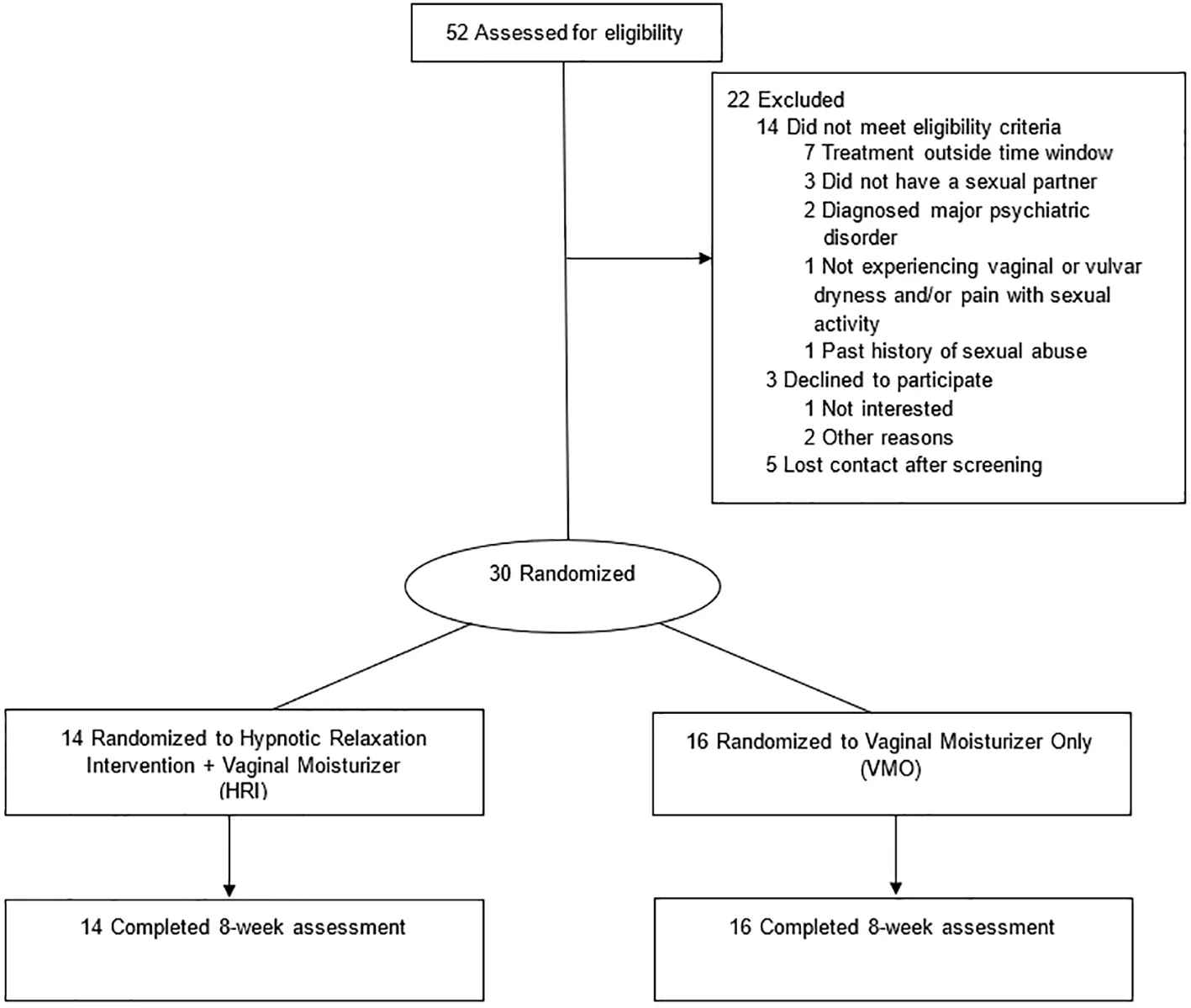
CONSORT flow diagram: women’s interventions for sexual health (WISH).

Of the 30 participants who were enrolled, 14 were randomized to the HRI arm and 16 were randomized to the VMO arm (see **Figure 1**). Most participants were white, identified as heterosexual, were married, and were from a higher sociodemographic background in terms of income and educational level (see **Table 1**). There were no statistically significant differences between study arms at baseline.

**Table 1:** Baseline characteristics of the participants.

Characteristic	Overall (*N* = 30)	HRI (*n* = 14)	VMO (*n* = 16)
Age, mean (SD)	55 (10.84)	52.79 (10.82)	56.94 (10.83)
Characteristic, participants, no. (%)			
Ethnicity			
Not Hispanic or Latino	30 (100%)	14 (100%)	16 (100%)
Race			
Black or African American	1 (3.3%)	0	1 (6.3%)
White	28 (93.3%)	14 (100%)	14 (87.5%)
More than one race	1 (3.3%)	0	1 (6.3%)
Marital status			
Married	29 (96.7%)	14 (100%)	15 (93.8%)
Single, living with partner	1 (3.3%)	0	1 (6.3%)
Sexual orientation			
Straight (heterosexual)	28 (93.3%)	14 (100%)	14 (87.5%)
Lesbian	0	0	1 (6.3%)
Bisexual	0	0	1 (6.3%)
Education level			
High school graduate or GED	1 (3.3%)	0 (0%)	1 (6.3%)
Some college	4 (13.3%)	3 (21.4%)	1 (6.3%)
Undergraduate degree	9 (30.0%)	5 (35.7%)	4 (25%)
Master’s degree	15 (50.0%)	6 (42.9%)	9 (56.3%)
Doctorate	1 (3.3%)	0	1 (6.3%)
Employment status			
Working full-time	20 (66.7%)	9 (64.3%)	11 (68.7%)
Working part-time	6 (20.0%)	2 (14.2%)	4 (25%)
Retired	4 (13.3%)	3 (21.4%)	1 (6.3%)
Annual household income			
$50,000–$74,999	2 (6.7%)	0	2 (12.5%)
$75,000–$99,999	3 (10.0%)	1 (7.1%)	2 (12.5%)
$100,000 or more	24 (80.0%)	12 (85.8%)	12 (75.0%)
Prefer not to answer	1 (3.3%)	1 (7.1%)	0
Do you have difficulty paying for basics, like housing and food?			
No difficulty	28 (93.3%)	13 (92.9%)	15 (93.8%)
Some difficulty	2 (6.7%)	1 (7.1%)	1 (6.3%)
Cancer stage at time of diagnosis			
Stage I	15 (50.0%)	7 (50.0%)	8 (50.0%)
Stage II	13 (43.3%)	6 (42.9%)	7 (43.8%)
Stage III	2 (6.7%)	1 (7.1%)	1 (6.3%)
In the past 30 days, did you have any type of sexual activity?			
Yes	25 (83.3%)	13 (92.9%)	12 (75%)
No	5 (16.7%)	1 (7.1%)	4 (25%)
Currently on adjuvant endocrine therapy?			
Yes	26 (86.7%)	13 (92.9%)	13 (81.3%)
No	4 (13.3%)	1 (7.1%)	3 (18.7%)

Due to rounding, totals may not add up to 100%. There were no significant differences between groups at baseline.

### 3.1 Feasibility

A total of 52 breast cancer survivors were screened for eligibility. Thirty-six (69%) were eligible, and of those, 30 consented to participate (83%) (accrual), which met the *a priori* benchmark of ≥60% accrual rate. All 30 participants (100%) completed the study and provided data through week 8 (retention, see **Figure 1**), which met the *a priori* benchmark of ≥80% retention rate. Twenty-seven participants (90%) used vaginal moisturizer as instructed (Adherence). In the HRI arm, adherence to hypnosis was 92%. These adherence rates met the *a priori* benchmark of an ≥80% adherence rate for each intervention.

A total of 25 AEs were reported. There were no significant differences between arms on the number, severity, or relationship of AEs. Seventeen of the 25 AEs were unrelated to the interventions. Five AEs were moderate and possibly related to vaginal moisturizer use (urinary tract infection = 5), and three AEs were mild and possibly related to vaginal moisturizer use (vaginal discharge = 2, abdominal pain = 1). No serious AEs were reported.

### 3.2 Preliminary intervention effects

Baseline outcomes for all exploratory measures (FSFI, SexFS V2, BITS) were not significantly different between groups. For both groups, the total FSFI scores at baseline fell below the clinically established cutoff for this measure of 26.55, indicating sexual dysfunction, and the FSFI desire subscale scores were below 3.3, indicating low desire (24). In both study arms, large effects were found for improved lubrication, *d* = 1.50 [confidence interval (95% CI): 0.97, 2.02], and a reduction in pain, *d* = 0.81 (95% CI: 0.39, 1.22) on the FSFI (see **Table 2**). Both arms showed improvements for all measures from baseline to week 8. Regarding differences between arms, there was a small effect on the change difference in the SexFS V2 interest, *d* = 0.37 (95% CI: −1.09, 0.36) favoring the HRI arm (see **Tables 3**, **4**). On the BITS, both groups improved over 8 weeks, and there was a moderate effect on the difference in change between groups, *d* = 0.42 (95% CI: 0.31, 1.15) favoring the HRI arm (see **Table 5**).

**Table 2:** WISH FSFI, PROMIS SexFS V2, and BITS within group effect sizes from baseline to week 8.

Variable	Full sample		HRI		VVA	
	Cohen’s *d* (baseline to week 8)	(95% CI)	Cohen’s *d* (baseline to week 8)	(95% CI)	Cohen’s *d* (baseline to week 8)	(95% CI)
FSFI: higher scores indicate better sexual function						
Desire	1.20	(0.72, 1.67)	1.15	(0.46, 1.82)	1.21	(0.55, 1.86)
Arousal	1.17	(0.70, 1.63)	1.11	(0.43, 1.77)	1.19	(0.53, 1.83)
Lubrication	1.50	(0.97, 2.02)	1.20	(0.49, 1.87)	1.85	(1.02, 2.66)
Orgasm	0.90	(0.46, 1.31)	0.69	(0.09, 1.26)	1.09	(0.46, 1.71)
Satisfaction	1.09	(0.63, 1.53)	0.91	(0.27, 1.53)	1.26	(0.58, 1.91)
Pain	0.81	(0.39, 1.22)	0.77	(0.16, 1.36)	0.82	(0.24, 1.38)
Total	1.52	(0.98, 2.05)	1.37	(0.62, 2.10)	1.63	(0.86, 2.37)
PROMIS SexFS V2: higher scores indicate better sexual function						
Satisfaction	0.92	(0.45, 1.39)	1.19	(0.45, 1.89)	0.80	(0.13, 1.44)
Interest	0.84	(0.42, 1.26)	1.10	(0.41, 1.75)	0.64	(0.09, 1.18)
Lubrication	1.75	(1.11, 2.37)	1.79	(0.88, 2.67)	1.63	(0.74, 2.50)
BITS: higher scores indicate worse body change stress						
	0.69	(0.28, 1.08)	1.00	(.34, 1.63)	0.50	(−0.03, 1.01)

FSFI, Female Sexual Function Inventory; PROMIS SexFS V2, PROMIS Sexual Function and Satisfaction V2; BITS, Breast Cancer Impact of Treatment Scale.

**Table 3:** WISH female sexual function inventory (FSFI) between-group differences: change in mean from baseline to week 8.

Scale	HRI (*n* = 14) Mean (SD)	VMO (*n* = 16) Mean (SD)	Differences between arms
			Effect size (95% CI)
FSFI: higher scores indicate better sexual function			
Desire, range = 1.2–6			
Baseline	2.06 (0.84)	2.03 (0.95)	
Week 8	3.17 (1.09)	3.08 (0.75)	
Change from baseline to week 8	1.11 (0.97)	1.05 (0.86)	0.07 (−0.65, 0.79)
Arousal, range = 0–6			
Baseline	2.51 (0.93)	2.12 (1.47)	
Week 8	3.88 (1.72)	3.45 (1.33)	
Change from baseline to week 8	1.37 (1.24)	1.33 (1.12)	0.03 (−0.68, 0.75)
Lubrication, range = 0–6			
Baseline	2.06 (1.26)	1.65 (1.33)	
Week 8	3.90 (1.71)	3.92 (1.76)	
Change from baseline to week 8	1.84 (1.55)	2.27 (1.22)	0.31 (−0.42, 1.03)
Orgasm, 0–6			
Baseline	2.83 (1.84)	2.58 (2.07)	
Week 8	3.80 (2.14)	3.90 (1.82)	
Change from baseline to week 8	0.97 (1.41)	1.33 (1.21)	0.27 (−0.45, 0.99)
Satisfaction, range = 0.8–6			
Baseline	2.86 (0.86)	2.65 (1.38)	
Week 8	4.06 (1.50)	3.88 (1.81)	
Change from baseline to week 8	1.20 (1.31)	1.25 (.99)	0.04 (−0.67, 0.76)
Pain, range = 0–6			
Baseline	2.26 (1.58)	1.80 (1.54)	
Week 8	3.54 (2.13)	3.20 (2.23)	
Change from baseline to week 8	1.29 (1.68)	1.40 (1.71)	0.07 (−0.65, 0.78)
Total, range = 2–36			
Baseline	14.56 (5.21)	12.82 (6.97)	
Week 8	22.35 (8.21)	21.44 (7.34)	
Change from baseline to week 8	7.79 (5.69)	8.63 (5.31)	0.15 (−0.57, 0.87)

There were no significant differences between groups at baseline.

**Table 4:** WISH PROMIS Sexual function and satisfaction V2 (PROMIS SexFS V2) between-group differences: change in mean from baseline to week 8.

PROMIS SexFS V2, *t*-scores have a mean of 50 and an SD of 10 in referent population. A score of 40 is 1 SD lower than the reference population. Higher scores indicate better sexual function				
Subscale	HRI (*n* = 14) Mean (SD)		VMO (*n* = 16) Mean (SD)	Difference between arms Effect size (95% CI)
Satisfaction				
Baseline		38.23 (5.49)	39.80 (4.19)	
Week 8		44.37 (8.14)	47.16 (8.26)	
Change from baseline to week 8		6.12 (5.17)	7.36 (9.23)	0.17 (−0.62, 0.95)
Interest				
Baseline		31.33 (9.01)	35.65 (7.51)	
Week 8		40.34 (11.10)	41.48 (7.78)	
Change from baseline to week 8		9.01 (8.22)	5.83 (9.06)	0.37 (−1.09, 0.36)
Lubrication				
Baseline		32.68 (7.11)	34.28 (6.23)	
Week 8		45.65 (9.74)	46.11 (8.63)	
Change from baseline to week 8		12.97 (7.25)	11.83 (7.24)	0.16 (−0.94, 0.63)

There were no significant differences between groups at baseline.

**Table 5:** WISH Breast Cancer Impact of Treatment Scale (BITS) between-group differences: change in mean from baseline to week 8.

BITS, range = 0–65: higher scores indicate worse body change stress			
Time	HRI (*n* = 14) Mean (SD)	VMO (*n* = 16) Mean (SD)	Difference between armsEffect size (95% CI)
Baseline	30.71 (14.91)	35.00 (16.54)	
Week 8	19.50 (14.85)	28.81 (16.67)	
Change from baseline to week 8	−11.21 (11.23)	−6.19 (12.37)	0.42 (−0.31, 1.15)

There were no significant differences between groups at baseline.

## 4 Discussion

This study confirms that HRI with vaginal moisturizer is a promising sexual health intervention in female breast cancer survivors. Using a multicomponent intervention, sexual health improved in all domains. This may be due to the use of vaginal moisturizer, which was administered in both study arms; however, HRI showed a trend toward an additional beneficial effect on body image and sexual desire with moderate (*d* = 0.42) and small (*d* = 0.37) effects favoring HRI. Consistent with the biopsychosocial framework of cancer-related sexual problems, addressing physiological and psychological sexual problems is essential, as these domains are interrelated (8, 13). For example, addressing painful sex is key to improving desire, as models of sexual response demonstrate that untreated sexual pain compromises the ability to improve interest (12, 15).

At baseline, both groups had sexual dysfunction with FSFI total scores below 26.55. While all domains of sexual function saw improvements, the large effects across the study arm were found on the FSFI for an improvement in lubrication, *d* = 1.5 (95% CI: 0.97, 2.02), and pain, *d* = 0.81 (95% CI: 0.39, 1.22). These results are consistent with a recent randomized controlled trial of non-hormonal vaginal moisturizers in breast cancer survivors which found improvements in lubrication and reduction in pain within 8 weeks of use (28). The vaginal moisturizer-only arm represented a rigorous and clinically relevant comparator, as moisturizers are guideline-recommended first-line therapy, yet are underutilized in breast cancer survivors and, therefore, constitute an active standard-of-care control (13). This pilot adds to the evidence showing that vaginal moisturizers are safe, acceptable, and beneficial.

However, although SexFS V2 lubrication scores improved for both groups after the intervention, scores remained two standard deviations below the reference group norm. These findings highlight that while short-term non-hormonal interventions can lead to clinically meaningful improvement, longer treatment duration or stepped, multimodal approaches may be required to more fully address vaginal dysfunction associated with prolonged estrogen deprivation (13). Future studies should evaluate extended intervention periods and combination strategies to determine their impact on achieving sustained, clinically normative sexual function. Additionally, this study included a non-hormonal bioadhesive vaginal moisturizer. Future research should compare different vaginal moisturizers, such as those containing hyaluronic acid.

This pilot study had several important strengths including the novel evaluation of a multicomponent intervention addressing both physical and psychological sexual problems, a rigorous control condition, and excellent retention rates. As a pilot study, this trial was designed to evaluate feasibility rather than efficacy, and the sample size was appropriate for that purpose. However, the sample was relatively homogeneous in terms of race and ethnicity, which is appropriate for a pilot study not designed to produce generalizable estimates. For the larger planned randomized controlled trial, we will aim to ensure a heterogeneous sample to bolster generalizability of the efficacy findings. A limitation of this study is the lack of blinding, which may have introduced expectancy and performance biases, particularly for self-reported outcomes. Although both groups received active interventions, which may reduce differential expectancy effects, unblinded staff interactions could still have influenced results. Additionally, the study was not powered to detect statistically significant between-group differences, and effect size estimates should therefore be interpreted with caution. Furthermore, our small sample size limited our ability to conduct meaningful subgroup analyses. Our planned trial aims to generate definitive efficacy data and allow for meaningful subgroup analyses, such as the influence of cancer stage on treatment outcomes of the interventions pilot-tested here.

## 5 Conclusion

This pilot randomized controlled trial demonstrated the feasibility of hypnotic relaxation and vaginal moisturizer for female breast cancer survivors experiencing sexual difficulties. The findings suggest a trend toward benefit for hypnotic relaxation coupled with vaginal moisturizer (applied vulvovaginally), particularly for improving body image. A larger, adequately powered study is needed to evaluate this intervention for efficacy.

## Data Availability

The datasets presented in this study can be found in online repositories. The names of the repository/repositories and accession number(s) can be found below: https://clinicaltrials.gov/study/NCT05692960.
